# Extra Supplement.—The Nursing Mirror

**Published:** 1894-01-27

**Authors:** 


"The Hospital" Nursing Mirror.

Being the Extra Nursing Supplement of "The Hospital" Newspaper.

[Contributions for this Supplement should be addressed to the Editor, The Hospital, 428, Strand, London, W.C., and should have the word "Nursing" plainly written in left-hand top corner of the envelope.]

### News from the Nursing World.

### WESTMINSTER HOSPITAL SAMARITAN FUND.

Her Royal Highness the Duchess of Albany has kindly consented to be present at Mrs. Calverley Bewicke's Shakesperian dramatic recital in aid of the Samaritan Fund of the Westminster Hospital, which is arranged to take place at the Queen Anne's Mansion Theatre at half-past three on Tuesday, February 20th.

### AFTER-CARE ASSOCIATION.

At the annual meeting of this association, reported in last week's "Mirror," a suggestion to obtain subscriptions from Boards of Guardians was opposed by Mrs. Shaen, a member of the Kensington Board. Such opposition is unaccountable, seeing that the law provides that Guardians may, and therefore being intelligent should, support associations of this kind in this way. Our experience convinces us that no better money is spent by Boards of Guardians than that which is wisely contributed by way of subscriptions to existing institutions, the work of which tends materially to relieve the rates. We are surprised that anyone should oppose a subscription to this association. When the opposition comes from a lady, who is also a member of an important Board of Guardians, the matter is serious enough to warrant inquiry, with a view to the inculcation of a juster perception of the duties of Poor-Law Guardians in matters of this kind. We shall be glad to hear from Mrs. Shaen, whose action will no doubt excite inquiry amongst the ratepayers of Kensington.

### HELPS HOMEWARDS.

"Well, Mrs. Brown," said the minister's wife, "and how do you like the new nurse?" "Sure she's a wonderful comfort, ma'am, and it's so cheery to 'ave a person as wants ye to live, about ye whiles yer ailing." "I am glad you like our Queen's nurse; some of the ladies thought you mightn't care for her after being used to old Mrs. Jones so long." "Bless yer heart," interrupted Mrs. Brown, with energy, "we ain't such fools as not to know the difference between a lady as wants yer to get better and a easy-goin' old creeter like Mrs. Jones, as knows nought 'cept how dying folks can be *helped home* quick and laid out 'ansome."

### REPEATED WARNINGS.

Typhus fever has made its appearance at Thurso, and amongst its numerous victims one nurse has died, whilst a doctor and another nurse are still ill. The available hospital with four beds is quite inadequate to meet this sad emergency, and the erection of a larger one is again under discussion. In The Hospital of October 14th we noted that the authorities were then in favour of building a new hospital, "the old one being on an insanitary site, and of inadequate dimensions." Typhoid fever was present at that time, and now, three months later, a visitation of typhus has occurred, and the sick poor remain still unprovided for. Are the responsible officials awaiting a third and even more disastrous reminder of their duty to their neighbour?

### THE R.B.N.A. AND COERCION.

We regret to see that the Council of the R.B.N.A. note with approval that the responsible authorities at the Sussex County Hospital at Brighton are giving countenance to a system which, in plain English, openly sanctions bribery. A premium of £5 is offered to each nurse who becomes a member of the R.B.N.A. at the end of her three years' training. She pays for this training, but can get £5 back at its conclusion on this one condition only. It is a little commercial scheme planned on the same principle (?) as that of the small shopkeepers who advertise that they will give away a teapot to each purchaser of a certain quantity of tea! At rich St. Bartholomew's the governing body appear to be taking a more economical course. Instead of rewards, they are prepared to mete out fines! Theirs will be indeed a reign of terror. Failure on the part of a nurse to "give satisfaction" to a certain number of individuals is to be fined to the extent of a current quarter's wages. Surely such regulations savour more of the dark ages than of the nineteenth century! Have the Governors of these two institutions neither courage nor common sense sufficient to protest against this shameless parade of coercion and bribery? The general public, however, has an equal interest in the funds of charitable institutions, and the gentlemen morally responsible for the management thereof will have to justify these high-handed measures in the eyes of all honourable Englishmen. The times are past for each man to do right in his own eyes without regarding his neighbour's opinion. No body of workers is more sure of protection and justice than the nurses of to-day, whether they be "training" or "trained." It is also probable that amongst the few members newly enrolled by the R.B.N.A. someone may prove individually strong enough to enter a protest against the treatment of probationers on Reformatory lines. The Matron of the Royal Free Hospital, who has been "proposed" during a considerable period, may, when her election to the Council becomes an accepted fact, show her associates that she has by no means forgotten how to exercise the large-hearted spirit of charity and justice which distinguishes King's College Hospital, where, as "Sister Henrietta,' Miss Wedgewood did many years of valued and valuable service.

### AN OVERFLOW.

The "overflow" treat given by the *Pall Mall Budget* in the North-Eastern Hospital for Children, on January 19th, was a decided success. The little guests, who assembled in the Out-Patient Hall, certainly had no cause to complain of the entertainment provided for them. Besides the solid gratification of a "surprise" bag for each small visitor, which contained gifts, eatable and otherwise, comic songs and glees were the order of the evening. The singing of Mr. Delamere was specially appreciated by the audience, about fifty of whom were out-patients, and the rest were gathered from among the poorest inhabitants of the neighbourhood by Mr. C. L. Boyer and his aides-de-camp of the Drift Mission. Among those present were the Rev. W. G. Morcom, who opened the entertainment with a kindly speech to the little guests; and Miss Glenton-Kerr, to whom is due the successful organisation of the entertainment, for which she was heartily cheered by those for whom she had arranged it. Not the least pleasant feature of the evening was the singing of the children themselves, led by Mr. Boyer. It was quite wonderful to hear how well, without any accompaniment, they kept time and tune, and it was also delightful to notice how much they enjoyed the sound of their own voices.

### A SCOTCH CO-OPERATION.

THE Glasgow and West of Scotland Co-operation for Trained Nurses has held its first annual meeting. It purports to be on the lines of the ably-managed Co-operation which has proved such a success at 8, New Cavendish Street, London, the nurses taking their own fees and paying 7½ per cent. on them towards office expenses. The Scotch nurses demand lower fees than the London ones, so their earnings must be reckoned at a lesser average. A large number of names are associated with the Glasgow Co-operation, which seems likely to be well supported by doctors, as well as of general benefit to the public. The rules include one which is certainly commendable, for we read: "The committee reserve the power to appoint a uniform to be worn by nurses while on duty." We should suggest that the sooner they exercise their power instead of reserving it the better. The Gainsborough and sailor hats, the almost invisible bonnets, and unbecoming toques, which are the outward and visible signs of nurses who are "working on their own account," are as unpopular with doctors and patients as the high sleeves, rough hair, and draggle-tailed skirts favoured by some members of the nursing profession.

### PROPOSED PROGRESS AT TIVERTON.

ATTENTION has been drawn to the necessity for securing an infectious hospital at Tiverton, and several cases have been mentioned where disease has spread to surrounding districts, because no proper system of isolation was enforced. The adoption of the Notification of Diseases Act has also been advocated in Tiverton, and it is to be hoped that steps will be taken without delay to secure nursing, isolation, and thorough disinfection, and thus promptly stamp out a danger which certainly is unnecessary and preventable.

### BETTER LATE THAN NEVER.

PROVIDENCE, in the form of the Local Government Board, has at last befriended the sick paupers in Knaresborough Workhouse. It seems that during the last four or five years the arrangements have been known to be unsatisfactory, and the officials have ruled with a rod of iron the sick and destitute patients. The lady visitors, who can do so much to brighten the lives of the unfortunate, have apparently ceased to visit the inmates at Knaresborough Workhouse because of the discourtesy shown to them by the officials. The Guardians seem well acquainted with the existing abuses which the Local Government Board devoted four days to investigating, and the dismissal of the "harsh-mannered" master and matron are the practical results. The medical officer, who has been in the habit of delegating his duties to an unqualified assistant, has been asked to resign. Probably sufficient light has been now shed on existing evils to ensure their soon being put down permanently.

### QUEEN'S NURSES.

ENCOURAGED by the success which has attended the work of Queen's Nurses in other districts the inhabitants of Earls Barton have raised funds to secure the benefit of a fully-trained nurse for their own sick poor. A successful meeting to promote this measure was recently, combined with a tea party, held in the Board school-room. Four hundred people were present, and listened with much interest to an address from Mrs. Bell, who, with advocacy of the views of the County Council on health-lecturing, also combined lucid explanations of the advantages to be derived from the permanent establishment in districts of women fully trained and experienced in nursing the sick poor in their own homes. Of course, Mrs. Bell's position as a certificated nurse and lecturer gave weight to her words, and it would be well if her advice were generally followed. Until the necessity for experience and thorough training in district work is universally understood, well-meaning but misguided philanthropists will continue to spread over the country, under the misnomer of "nurses," a class of persons whose knowledge of "charing" has been veneered with the superficial accomplishment of "the three months' course."

### NURSES AT THE ROTUNDA.

THE new auxiliary building in connection with the Rotunda Hospital at Dublin will provide proper accommodation for the nurses as well as various much-needed conveniences for the patients. For some months past over twenty nurses and probationers have been quartered in a small concert-room, and the board-room has formed their refectory. This by no means ideal arrangement was the best which the committee could manage with the limited space at their command. Previously nurses, pupils, and wardmaids were obliged to sleep in the same wards as the patients. Considering the high reputation which this hospital has for training pupils in its special branch of nursing, it is certainly time that suitable sleeping accommodation was provided for the nurses and students. Amongst the latter there are natives of various countries, including Parsees.

### HOSPITAL FOR SICK CHILDREN.

THE Lady Superintendent of the Hospital for Sick Children, Great Ormond Street, having retired on account of ill-health, the post is filled temporarily by Miss Power, who has had charge of Cromwell House, the Convalescent Home connected with the Children's Hospital. Miss Power, who is a daughter of the well-known author of "The Oiled Feather," &c., was trained at King's College Hospital, and she has already shown marked ability as an administrator, and also in securing the interest of visitors for the little ones under her care.

### SHORT ITEMS.

A CONCERT in aid of the Home for Consumptive Women was held at the Queen's Hall, on 16th inst. There was a large attendance, so the funds of the charity ought to benefit considerably from the results of this pleasant entertainment.—A small hospital has been lately opened at Thames Ditton, and the number of beds, at present five in number, can be added to if desired. The cost of the building, £800, has been already raised. The opening ceremony was performed by Miss Went.—A district nurse has been appointed at Felton. She commenced work with the New Year. —The Western Ophthalmic Hospital, in Marylebone Road, is now open on Thursday evenings from six p.m. for the convenience of out-patients who cannot leave their work at other hours.

### On Nursing the Nervous and Insane.

By T. DUNCAN GREENLEES, M.B.Edin., Medical Superintendent, Grahams Town Asylum, South Africa.

### III.—USES OF ELECTRICITY IN NERVOUS DISEASES.

ELECTRICITY is of use in all diseases of the nervous and muscular systems where the functions are not completely obliterated or destroyed by organic tissue change. Where there is such a change in the tissue—whether nervous or muscular—as to prevent the proper flow of neural impulses from the central to the peripheral organs, then electricity in the treatment of disease is of little benefit. We can, however, in these cases imitate nature. Thus, after apoplexy sometimes one or other of the limbs are paralysed; this is due to an arrest in the nerve current from the central organs, the brain to the muscle, or groups of muscles affected. Now in this case the muscles are healthy, the nerves supplying these muscles are likewise healthy; it is only the brain that is diseased. If nothing is done the muscles, from disuse, waste and become atrophied. To induce muscular contraction, and thereby imitate nature, an electric current is passed along the healthy nerves; this stimulates the muscles to contract, and, for the time at least, their functions are artificially restored. If this treatment is persevered in, the paralysed limb not infrequently regains its wonted plumpness, and if the brain in time recovers its health, the normal functions return to the limb likewise.

Faradism suffices for all cases where the paralysis is due to a central lesion, such as the example given when the paralysis is caused by disease of the brain. If the disease, however, is due to some peripheral cause, such as chronic rheumatism, lead poisoning, or writer's cramp, then galvanism will be required.

To reach the nerves, certain points, called motor points, have been mapped out on the surface of the body, these represent the inlet and outlet of the different nerves. In applying electricity the electrodes, or poles, as they are called, are placed over the motor points of whatever muscle, or group of muscles, require stimulating. The poles of an electric machine are termed positive and negative; if an ascending current is desired, the positive pole is placed over the outlet and the negative pole over the inlet of the nerve; while when a descending current is indicated the position of the poles is reversed.

These poles or electrodes are made of different shapes to suit different portions of the body, and their surface is generally moistened with a saline solution before application, to enable the current to reach the nerve with greater ease.

There are certain dangers to be avoided and cautions to be observed in the application of electricity to the treatment of disease. When first applied a weak current should always be used, and, if necessary, gradually increased in strength.

The séance, or sitting, should at first not last more than a few minutes; afterwards, as the patient gets accustomed to the applications, the sittings may be prolonged to half an hour, or even longer.

The cases for electric treatment should be carefully selected by a medical man, and no nurse should attempt this treatment without the advise and supervision of the medical attendant. Injury of a permanent character has followed the injudicious application of electricity, or the sudden application of a very strong current in persons peculiarly susceptible to its influence. Blindness has been produced by a current passed through the head, and vertigo and fainting occur when the current has been made too strong. Muscle and nerve functions have been permanently abolished by strong currents or prolonged séances, more especially where the treatment is persisted in until exhaustion results. This should never be done; contraction can be produced by stimulation of a muscle, but if the stimulation is too powerful and long continued, the muscle becomes exhausted and its power of contractibity is destroyed.

In mental diseases electricity is not without its use in stuporous cases, and those cases of melancholia that seem simply to require rousing up, the application of a weak current through the head—the positive pole being placed over the forehead and the negative to the nape of the neck—sometimes produces very satisfactory results. Again, in some cases of moral insanity the electric current has a beneficial effect as a moral deterrent, although its general uses cannot be reccommended for such patients; and, indeed, in insanity there are really very few cases where this treatment alone has produced such results, as might almost be expected from knowledge of its powerful and beneficial effects in nervous diseases.

B. MASSAGE.—This form of treatment was known to the ancients, and has recently, namely, during the past 10 or 15 years, been revived. Like most of the modern methods, it has its enthusiastic votaries, who claim for it a specific action in all diseases, and is said by them to be the panacea for all the ills that flesh is heir to. This is a curious fact in modern scientific medicine; that there is a continual searching for something new, and, when found, it is applied to the treatment of every disease. Enthusiasm regarding the good qualities of a drug blinds common sense, and haste is made to see and to note imaginary benefits arising from its use. After a time the enthusiasm cools down, and the treatment is relegated to the things of the past, unless it is really proved of some use; then it finds its position in one of the many sub-divisions of the extensive science of therapeutics. So with massage: formerly looked upon as a "cure-all," it is now beginning to occupy a more definite, although more limited, position in the treatment of disease, especially in neurotic and hysterical cases, as well as in chronic rheumatism and constipation from atomy of the intestinal or abdominal walls.

The word massage means to rub or to press softly, but the process includes kneading the muscles in the same way as the baker kneads the dough.*

* Massage (μάσσω, to knead). The methodical kneading, manipulation, rubbing, and beating of the limbs and trunk.—Mayne's "Medical Vocabulary."

### Appointments.

CARMARTHEN JOINT COUNTIES LUNATIC ASYLUM.—Miss Mary Thomas has been elected Matron of this asylum. She was for a short time attendant at Bridgend Lunatic Asylum, housekeeper in a private family, and manageress of a house of business.

### Minor Appointments.

BELFAST LUNATIC ASYLUM.—Miss Reid Stewart has been appointed Head Nurse at this asylum. She was trained at the Sterling District Asylum.

CEYLON NURSES' ASSOCIATION.—Miss Ellen Bumstead has been accepted on the staff of this association. She was trained at King's College Hospital, and in midwifery at Whitechapel Union Infirmary. Miss Bumstead was head nurse at Dover Infirmary, and subsequently held a similar post at Cuckfield Infirmary. The good wishes of many fellow workers and old patients accompany her to her new work.

### Presentation.

MISS WOODCOCK, the Director of the Hollond Nursing Institute, Nice, was presented on New Year's Day with a brass travelling clock, in red morocco case, by the Superintendents of the various branch homes.

### District Nursing.

### IV.—DISTRICT HYGIENE.

### Traditions Unwillingly Forsaken.

Good air and proper food are two essentials of health generally conspicuous by their absence from the homes of the poor. Tradition and prejudice have more hold over the people than is generally recognised; it is extremely difficult to make them see why they should depart from the ways of their forefathers in their domestic arrangements. There is an apathy and fatalism amongst them which baffles the would-be reformer at every turn, and this is sometimes urged as an excuse for not endeavouring to raise their condition. The real source of the evil is distinctly traceable to absolute ignorance of the most elementary laws of health—they have never been taught, never been made to understand, and "knowledge is of things we see."

### Instruction of the Young.

Much is being done in the right direction in these days to bring home these laws of health. Teaching the children is the thin end of a wedge that will open the way to better things. "Health Missioners" who talk to wives and mother in simple, homely language, and teach them elemental facts as regards themselves, their children, and their homes, are also pioneers. District nurses can help largely in the work in a quiet, unobtrusive manner. They know only too well exactly how the people live, and therefore can be really practical. It is no use to talk of sunny bed-rooms and children kept from playing in the damp, when too often one room serves as bed-room, sitting-room and kitchen for a whole family in a court so narrow that sunlight has never penetrated into it since it was built, while the children's playground is either the court in front, or a well-like yard at the back of the house. In the country and towns, where space is not so valuable, the dwelling places are lower in rent, and more rooms can be taken by a family; but the majority of people, even among those interested in questions of reform, have very little, if any, idea of the homes of the poor, and the rents demanded for them, in many parts of the metropolis and large cities and towns.

### Making the Best of It.

District nurses must take things as they find them—their duty consists in never ceasing to urge the possibility of better ways. They cannot alter crowded alleys or damp, ill-ventilated cottages, but they can bring cleanliness and hints to improve many defects, and can always, in London at any rate, urge removal to the better accommodation provided in so many quarters.

### "The Lord's Will."

Increased knowledge is dispelling the devil's doctrine so valiantly resisted by Charles Kingsley and put into a nutshell by Tennyson, who makes the bereaved parent say—

"I thowt 'twur the will o' the Lord,
But Miss Annie, she säaid it wur draains."

Make a father and mother really understand that smelling sinks, cesspools, and drains mean lowered vitality, readiness to take diseases, and too often death to themselves and their children as surely as fire burns, or sucking matches poisons them, and they cannot go on in dull contentment with their surroundings. District nurses deal with the homes of the poor, and with the poor themselves, but before attempting to teach they must be educated.

### Practical Hygiene.

Hygiene is a science itself, and only a few of the fundamental principles can be taught without a regular course of instruction. But every district nurse should have a few simple practical lectures on drainage and its objects, with its appliances of traps, sinks, cisterns, &c., the laws which govern ventilation, the storage of water and the sources whence it comes, the nature of nuisances and their legal remedies, overcrowding and its effects, and to whom she should apply when she finds anything wrong.

### The Sanitary Institute.

To those who live in or near London a visit to the Sanitary Institute and Parkes Museum, Margaret Street, W., will prove most instructive, as there can be seen every modern improvement in drains, systems of ventilation, &c., and contrasted with them the defective methods, which alone constitutes a valuable object lesson. These are all explained to any wishing to understand the subject.

When a nurse understands the "reason why," she will not be content to leave her patient in a room where a gaily-decorated board hermetically seals the entrance to the chimney. The window, duly open during the day, is almost certain to be closed at night, and the patient sleeps, or does *not* sleep, in an atmosphere laden with carbonic acid gas and organic matter. The removal of the board may not be enough; knowledge of ventilation laws will suggest a lighted lamp being placed in the fireplace, which, establishing a current, ventilates the room without undue draught, and prevents the "down-draught" so often the excuse for blocking up a chimney. This is only one of many instances where increased knowledge is useful.

### Knowledge Practically Applied.

A district nurse should be able to tell if the sanitary arrangements of a house are in order; whether one cistern supplies to the drinking water and for flushing purposes; if the flush of water in a closet is sufficient; if the rain pipes discharge into the open, and also the overflow pipes from cisterns; if sink pipes run directly into drains or discharge in the open; and she should be able to give practical instruction as to cleaning furred and stained closet pans. Country nurses must be alive to the dangers from the proximity of cesspools and wells, surface wells and shallow springs, foul manure heaps, pigstyes, neglected privies, &c. Knowledge of facts will lead to practical hints to prevent mischief arising from ignorance of these evils.

### The Rights of Landlords.

But a word of warning must be given, lest in their zeal for improvement nurses forget they are dealing not with the patient's property, but with the landlord's, and undue interference will most probably lead to the ejectment of the tenants they are seeking to serve. Nurses must not be amateur sanitary inspectors. If they come to be regarded in that light they will do more harm than good, and their own work will suffer. People will be shy of admitting them into their homes, and thus they will defeat their own ends. Much can be done by tact and observation. A nurse who knows what to look for will soon grasp the sanitary condition of a dwelling. While emptying the slop pail she can draw her own conclusions as to the cleanliness of the closet, the flush of water obtainable, the state of the back yard, dustbin, &c. And it may be remarked, by the way, that it is a wise rule to empty the slops personally at least once in every house. Very often there are willing friends to run down with the pail "to save nurse," but an opportunity can be made to do this without giving rise to any suspicion of investigation on the nurse's part.

### Temporary or Permanent Evils.

If smells are noticed, it is easy to ascertain whether they are permanent or accidental, in the course of general conversation; so many facts can be elicited, casually as it were, with regard to the emptying of the dustbin, the cleanliness of the cistern, the condition of the yard, whether there are rats (often a sign of imperfect drains), &c.

The following list is issued from one of the sanitary aid committees of the Mansion House Council on the Dwellings of the Poor. Any complaints of the following may be sent to the hon. secretary: Bad smells from drains, bad smells from manure heaps, bad smells from dustbins, bad smells from water closets, bad smells from old rags and bones, bad smells from animals improperly kept, or any other cause, bad smells from broken gutters, pipes, or traps bad or insufficient water supply, holes in walls, ceilings, floors, or roofs, over crowding, unhealthy cellar dwellings, and damp and ill-paved yards.

These sanitary aid committees are invaluable to nurses working in the metropolitan area. The central office is 51, Imperial Buildings, Ludgate Circus, E.C. Any communication marked "confidential" is so treated, and therefore the nurse is not quoted when the case is investigated. By the agents of this association the responsible person is made to correct the evil.

Consultation with Responsible Authorities.

Nurses should acquaint themselves with the sanitary authorities in every place, as rules vary in different localities. In the country the medical officer of health for the district is usually the right member to consult if the nurse thinks anything is wrong. His knowledge of local arrangements will prevent her making mistakes in her efforts to do good.

While advocating the use of disinfectants for sinks, drains, &c., it must never be forgotten that hiding a bad smell by a powerful deodorant, such as chloride of lime or strong carbolic acid, is not remedying the evil; on the contrary, it is actually harmful, as it lulls people into a state of false security. A sink that smells when it is going to rain needs more drastic treatment than flushing with a disinfectant.

Another point to be remembered is that it is not the smells themselves that are harmful, but the causes which give rise to them. A house may be saturated with sewer gas, without much, if any, odour. The nurse's suspicions will be roused by the complaints of sickness, diarrhœa, and sore throats amongst the inmates. They will complain of unrefreshing sleep, headache, and nasty taste in the mouth on waking, constant weariness, loss of appetite, a tendency to boils, gathered fingers, &c. Wounds in such an atmosphere are slow in healing, and there is a marked liability to erysipelas. Investigation will generally lead to the discovery of defective drainage, and very often to dirty cisterns, also so constructed that the water can absorb sewer gas.

Overcrowding.

Nurses are frequently confronted by the problem of overcrowding. Too many persons occupying a room of given cubic space constitutes legal overcrowding, and this may be reported to the sanitary authorities.

But the constant domestic overcrowding that goes on every night is really more deadly, for it poisons the children, lays the foundation for phthisis and rickets, and strongly predisposes to bronchitis and pneumonia.

Avoidable Dangers.

There is terrible mortality amongst infants from suffocation in the night, and there is no reason why, after three weeks or a month, the child should not sleep in a basket, box, or cot by the mother's bed. This is law in Germany, and many a baby life would be saved if it were enforced in England.

Children should not sleep in the same bed as adults if it can possibly be avoided, nor too many together in one bed. Nurses can do much by improving the usual sleeping arrangements of many poor homes.

### Death in Our Ranks.

Miss Kate Lambert died on December 30th, of typhoid fever, at Sheffield General Infirmary, where she had been a Sister for five years. She had previously held the position of Sister of the Isolation Wards at St. Mary's Hospital, Paddington. By her death the infirmary loses a devoted worker, and one whose kindly disposition and unselfishness made her a favourite with patients and fellow-workers.

### Where to Go.

Hotel Metropole. — Annual banquet on Saturday, February 3rd, in aid of the funds of the French Hospital, Shaftesbury Avenue.

### Stray Visitors.

(By One of Themselves.)

Some subjects come forward for discussion which prove easy to deal with, and then to dismiss altogether. Others are less simply disposed of, and they return and demand fresh attention after a peremptory fashion. The admission of visitors to hospitals belongs to the latter class, and the question embraces more interests than appear on the surface. We are not speaking of visitors who come on any kind of business, nor yet of the friends of the patients, but purely of casual callers whose motives are certainly various.

Suppose, however, that anyone comes from unaffected curiosity, just to see what a hospital is like. There is nothing to object to in this, and if the first impression is a pleasant one the visit may be repeated with benefit to the individual and also eventually to the institution. But if an unpleasant idea is conceived, then assuredly the effort will not be repeated by a person who will retain the impression to the end of his days that he was treated as an intruder.

As long as hospitals are supported by a charitable section of the public, just so long will all members of the general public have a right to be visitors to the wards. At reasonable times and seasons, goes without saying, in this case.

We heard the subject pretty freely discussed in a drawing-room the other day, and one lady remarked, "I like going to the wards, but I really shall give it up; the nurses dislike visitors so much." Her friend swiftly rejoinded, "But don't the patients welcome you"? and the first speaker replied, sadly enough, "Yes, there is never any fear of discourtesy from them, but I can't face the nurses any longer."

Surely this should be an impossible state of things? When complaints are made by officials that no gifts are received for their patients it would often be well to inquire what reception is usually accorded to visitors. It is from them, from the friends of the nurses, or of the students, and also from a section of the general public, that generous offerings are received. Institutions are not like private houses from which invitations are issued ere an acquaintance can be formed. Often those rich and liberal supporters of hospitals whose names become a power in the land have dated their first interest in these places to "someone's" having made them welcome inside the walls.

People go to some of the largest and busiest buildings and guess from the manner of the porter at the gate that they may count on a courteous reception from every member of the staff. Again, at some little hospital or home where the work is neither urgent nor great, the shy visitor is stiffly bidden to wait till the clocks have all chimed the hour selected by a committee as the proper one for strangers to undertake a stroll through half-filled rooms. But neither manners nor a cordial reception can always be ensured by a gracious matron, for old traditions exist that surliness and goodness may fairly go hand in hand. Still, there is hope that the little leaven of courtesy may presently leaven the whole lump of uncouthness. Few indeed are the institutions in our present progressive age where bad manners are still in fashion, and some day we hope to say there are none.

However, the improvement has been steady during the last few years, and we may safely anticipate a time when the universal courtesy existent in many, shall be characteristic of all places nourished in the all-embracing name of charity. When that day comes we shall no longer hear strangers ask, in nervous dread of the answer, if it "is quite convenient" for them to enter the wards. Nor shall we notice the visitor hesitating on the threshold, lest a haughty stare from a lounger shall be the only reception accorded. All honour to steadfast workers who, merging themselves in their duties, never waste a single opportunity for arousing outside interest in their patients. Their success is greater than they will probably ever know, and the triumph over innate selfish instincts is not the least part of their victory. Rudeness is always of ignoble origin, and comes from ignorance, vanity, or selfishness, showing utter want of consideration for the feelings of others.

### Nursing in Argentina.

### SCHOOL FOR MALE AND FEMALE NURSES.

### Gratuitous Instruction.

The Municipality having formed a School for Nurses in connection with the Public Assistance, the object of which is to prepare persons desirous of devoting themselves to this career, so that they may obtain a diploma of efficiency entitling them to exercise their profession in hospitals and private houses. Male and female nurses are invited to enter this school, open on Tuesdays and Saturdays from eight to nine p.m., in the building of the Public Assistance. Doctors having assistants in their employ, or acquainted with nurses, are requested to advise them to profit by the benefits this gratuitous instruction will afford them, and to register their names, if bearing diplomas.

### The School Register.

At the same time this school will serve as a centre of information, and a place where doctors may have every facility for obtaining at any moment skilful persons to undertake the care of their patients, and where nurses may obtain positions in hospitals or in private houses.

The conditions of admissibility for male or female nurses are the following: 1. To be at least twenty years old, healthy, and active. 2. To know how to read, write, and keep accounts. 3. To unite the conditions of morality, respectability, and neatness.

### Special Classes.

On Saturdays, from eight to nine p.m., the special course of lectures on the means of rendering "First Aid in Cases of Accidents" will be given. All persons wishing to acquire this knowledge may attend.

### Foreign Diplomas.

Persons holding foreign diplomas can have their title made valid, and registered in the book for that purpose, on presenting their certificate, or by undergoing an examination to prove their eligibility.

The Public Assistance will make known, by means of circulars, the number of persons who have received diplomas, and will give information and recommend the male or female nurse according to his or her ability.

Later on the teaching will be amplified so as to extend diplomas to specialties, such as accouchements, litters (or stretchers), bathers, masseurs, keepers of the insane or of children, &c.

### Service of Nurses.

The public can apply at any hour for male or female nurses at the office of Public Assistance.

Any person asking for a male or female nurse must pay for a ticket, which will be delivered to them with a signed receipt, in which the name of the assistant, his domicile, and nationality will be stated, as well as the direction he is going to, and whether the malady is contagious or not. The Direction of Public Assistance will at once send for the nurse designated, who will either follow the applicant at once or go to him later on.

The demands for practitioners, masseurs, midwives, pedicures, persons to apply blisters, caustics, &c., will be duly attended to.

Applications of nurses, male or female, for permanent positions in hospitals are attended to gratuitously, notice being given at the school on Tuesdays or Saturdays from eight to nine p.m. The answer will be given the same evening, whether or no there are vacancies.

In the event of the nurse having to go outside the city the expenses of the journey to and fro will be defrayed without prejudice to the regular remuneration.

The public must give notice, and pay the nurse up to the date of the ticket. Those who do not settle with the assistants will have their names put on a list, to prevent others being sent to them. The money received for a ticket cannot be returned on any pretext; the person interested can demand another nurse within the twenty-four hours if the one designed should not suit. Neither will directions be given to any nurse until the price of the corresponding ticket shall have been paid.

Minimum pay received at present by male or female nurses from the public:—

For contagious or chronic diseases from $3 to $4 per day and $60 per month. Nurses engaged by the month must be paid the entire month whether occupied or not, and in all cases they must have six hours' rest per day.

For contagious and acute maladies $4 to $5 per day and $90 per month.

The special attendance on accouchments and care of the insane, &c., is paid the same as cases of acute diseases.

When the service of nurses has not a sufficient number of those holding diplomas on the list, the most experienced among the students will be recommended, and in such cases the public will permit their attendance at the theoretical classes on Tuesdays and Saturdays from eight to nine p.m.

It will be stated on the ticket that the student (male or female) will be under the obligation of attending these lectures Tuesdays and Saturdays from eight to nine p.m.

### Obligatory Rules.

Nurses holding national diplomas, or foreign diplomas that have been ratified, who wish to be recommended to the public by the Public Assistance, must register their names at the beginning of every year on the list of the unoccupied, so as to be recommended according to the order of registration. They must state the special branch they devote themselves to, the remuneration they expect for night or day service, the languages they speak, and the date of their first assistance, &c.

Midwives who desire it may be recommended as monthly nurses, always provided they submit to the same conditions as those holding national diplomas.

Registered practitioners must be practically acquainted with the programme of the school.

Those inscribed should, in their own interest, co-operate to regularise the service of the male and female nurses, so as to make it as expeditious as possible; thus, the person on the list should proceed at once (within three hours) to the house indicated, as he can there refuse the conditions or remuneration proposed to him, and in that case return immediately to the Public Assistance, and inform the employé on duty, so that another nurse may be sent without delay.

Nurses must always give notice, should any personal or family impediment prevent them from accepting a place during the day, as they will have to be suspended from the list until the obstacle is removed, otherwise the desired rapidity in placing nurses would be interfered with. If a nurse should repeatedly cause such interruptions, or should he (or she) leave the patient without justifiable cause, at short notice, he (or she) will be struck off the list of the service. When a ticket is renewed (or receives extension), the person inscribed therein must ask for a copy of the list of the unoccupied, and on renewing the registration must at the expiration of his term of service bring the ticket of service, or, failing this, the medical certificate, of recent date. If all these conditions cannot be fulfilled, or if a long period has elapsed since registration, a sum of $5 must be paid before they can be again placed on the list.

Nurses who have attended patients with contagious diseases must allow a week to pass before presenting themselves to be again inscribed on the list; if they do so before the time mentioned, or if they have carried contagion to another house, they will be suspended from the list for at least one month.

No nurse can be permitted to inscribe herself on the list who has been attacked by any ailment or infirmity, or who may be in an advanced state of pregnancy, nor those who may have received into their house anyone suffering from an infectious disorder.

### Women's Work.

### AS MEDICAL STUDENTS.

(BY A VISITOR.)

SHORTLY before the opening of the winter session it was my privilege to be shown over the London School of Medicine for Women in Handel Street, W.C., and to obtain a good deal of information as to the history of the school and the work that it has done. Interesting as it was to see the class-rooms, laboratories, and museums in which the women doctors of the future are educated, a much more vivid idea of their work was gained by a visit to the Royal Free Hospital in Gray's Inn Road.

This hospital is an institution which deserves well of the public for many reasons. Founded by the late Mr. William Marsden Sugden as a medical charity "in which destitution and disease should alone be the passport for obtaining free and instant relief," its history of good work since it was opened in 1828 is a history of which those who are connected with the hospital may well be proud. The hospital is also specially entitled to the regard of all friends of the movement for the medical education of women, for it was the first to afford lady students opportunities for clinical instruction. The London School of Medicine for Women was opened in 1874, and it then became necessary that a hospital should be found with 150 beds at least to admit students to its practice. This was essential to obtain recognition of the school by the examining bodies. It was not easy to find a hospital to countenance the new departure, and for a time the prospects were not of the brightest. Not until 1877 was the difficulty removed, and in that year the governors of the Royal Free Hospital consented to admit women students, and entered into an arrangement with the authorities of the School of Medicine for Women which has worked harmoniously, and has proved advantageous to both institutions.

It does not in the least detract from the credit which belongs to the Royal Free Hospital for its public-spirited action at this juncture to mention that it has reaped the reward of its courage. It is good for a hospital to be connected with a medical school, and moreover, the public is doubly indebted to an institution which combines with the work of treating the sick that of training the medical advisers of the future. At the present moment the Royal Free Hospital is greatly in need of funds to meet the expenses of alterations and additions to the buildings which have long been necessary. In their appeal to the public, the governors lay stress upon the connection that exists between the hospital and the School of Medicine for Women. They do well to bring this forward, and the fact is one to which the fullest weight should be given by those who believe it good that there should be women doctors, and who know that there is most important work for them to do.

The female medical student is the object of a certain curious interest to any one familiar with the life of the male student. An opportunity of seeing how the woman comports herself under similar conditions recently offered itself, and the writer gladly paid a visit to the Royal Free Hospital during working hours, and through the kindness of Mrs. Thorne, hon. secretary of the School of Medicine for Women, and a member of the Ladies' Visiting Committee of the hospital, saw for himself the woman student at her work. At midday the work in the casualty department begins, the room which has been temporarily allotted to this purpose being thronged with patients. Here was seen a student engaged in dressing a boy's burned hand; there, under the directions of the house surgeon, another was binding up a wound. In one corner a group of students were in earnest consultation over a case; in another, an old man, who had evidently got over the worst of his injuries, chatted merrily with those who ministered to him. The appliances in the casualty rooms appeared by no means as complete as those in corresponding departments of other hospitals. For instance, receivers, which are such obvious necessities, seemed either altogether absent or so limited in number that the bowls of disinfectants did double duty. They received the absorbent wool both after and before its contact with wounds, and, alas! the soiled dressings were scattered on benches and floor. Perhaps the need for a trained nurse to supervise these details is not yet acknowledged at the R.F.H., but her presence is evidently most desirable. The appearance of the wards and the cheerful faces of the patients testified to the care and attention bestowed on the work at this hospital, and it may be noted with regard to the students that the feeling of the patients is not only one of approval but gratitude.

The arrangements with regard to clinical instruction are very complete, and students do not begin their attendance at the hospital until the second year. Their first year at the hospital is devoted to minor surgery, and to clerking and dressing in the out-patient department. Thereafter the student takes duty in the wards under a member of the staff, first in the surgical and then in the medical ward. Each student is enabled to work for a term under each of the senior members of the staff. Moreover there are tutorial classes in various special branches of medicine and surgery which students are encouraged to attend. The hospital possesses a good pathological museum, of which Miss Aldrich Blake, M.B., is curator. The classes at the school are so arranged as to allow students, after the second year, to give practically the whole day to work in the hospital. Upon the clinical side of medical study the authorities of the London School of Medicine for Women lay the fullest stress, and therein they show a true appreciation of what medical study ought to be.

### The Real and Ideal Nurse.

WE all know the picture the word nurse evolves in the lay mind. The sick man on his bed, the gentle-voiced nurse stooping over him, smoothing the pillow, bathing the fevered head, shading the too fierce sunlight from his weary eyes. How deftly she moves about the room, how skilfully she applies the necessary remedies, how spotlessly neat and dainty is her appearance. How she shines on the gloomy horizon of the sick chamber as an angel of light and healing. How devoted she is to her patient, how utterly oblivious to all else. That is the ordinary sentimental sketch of the hospital nurse, but is it a true one? Most of us who know anything of the subject are fain to shake our heads sorrowfully and acknowledge it is very far from it, speaking more particularly of the nurse as we find her in large provincial hospitals. The private nurse may realize this ideal sketch little better, but not if we judge of her during her training. "Well," you say, "give us a description of the average hospital nurse as she really is." It is more easily said than done. To begin with, she is generally a mass of contradictions. She has high ideals, a vague sense of the nobility and self-sacrifice of a nurse's life, a real desire to be noble and to do great things; but, alas! a very unsatisfactory way of performing little things which fall to her daily lot. Not that she always does the small duties badly; far from it, the average nurse usually takes an honest pride in these little things. But in such matters as preserving a cheerful, contented spirit, in fighting down the inclination to complain of petty annoyances, constantly occurring in a large establishment, she fails lamentably. Grumbling is a rampant vice in nearly all large hospitals. "Well," you say, "perhaps in most hospitals the nurses have some good reason to grumble." Possibly it is so, but were everything made as comfortable as possible, the nurses would still find cause for complaint. They are such trivial things, too, of which the average probationer makes a grievance. The doctor has passed her over in one of his clinical lectures, and addressed his remarks to a junior companion; the Sister has given one of her dressings to another nurse; the Matron has found fault with her cap. Such contemptible trifles to exercise the mind of a high-minded woman, but I am afraid it is these little things that form the nurse's trials. Is there no remedy for this state of things? Most nurses have a high ideal at heart, and grieve to think how far short of it they fall. They should cultivate that ideal, and then strive to bring their outward lives more into conformity with it. This in great measure may be done by widening their narrow circle of interests. A nurse's life is an engrossing, sometimes an exciting, one, of a nature to absorb her utterly to the exclusion of other interests. But is this good? She passes the greater part of her time in the wards, and "off duty" is usually spent talking "ward," which inevitably tends to develop into "ward gossip," and of course into grumbling. If on the contrary a nurse would, the moment she leaves the ward, throw it aside, so to speak, and devote herself to something different, she would go back to her work braced up and ready for duty. If she is literary let her read, not merely dip into a magazine now and then, but really read, try to keep up with the current literature of the day, and not forget the masterpieces of the past. If she is artistic let her take sketch-book and palette and seek nature. If her tastes are antiquarian, let her buy a guide-book, and sally forth to explore the neighbourhood. It matters not what it is, so that it saves her from sinking to the level of a mere nursing machine, without a thought beyond her daily round of work. Nursing is a noble profession, but it is not elevated by its votaries refusing to heed all else that is good. "But," you say, "this is all very well, but how can a poor, hard-worked nurse, with at most two hours a day 'off duty,' find time for all these things?" Do we not always find time for the little gossip, the spiteful tittle-tattle, the querulous complaints? Then why not for better things?

If in each of our large provincial hospitals a nurses' social club were formed, the general tone would be doubtless much improved. Such a club might meet once a week in the nurses' sitting-room when the working hours were over. It would need no elaborate organization, simply a social gathering of the nursing staff. Music, recitations, needlework, round games (occasionally, perhaps, theatricals), would pass away a couple of hours pleasantly, and in most hospitals the authorities would gladly countenance such meetings. A widening of interests would go far to destroy the present state of things. Above all, every nurse who would "be good and do good" must preserve her ideals; she must set constantly before her the One Great Exemplar, of whose life her own, if ever so faintly, is still a copy, and each and every day she must

> "Ask God to give her skill in comfort's art,
> That she may consecrated be, and set apart
> Unto a life of sympathy."

So only can she hope to walk worthy of the vocation wherein she is called.

Max Pireau.

### Notes and Queries.

### Queries.

(1) *Sanitation.*—Can anyone tell me how a provincial nurse can study for the diploma of the Sanitary Institute?—*Country Bumpkin.*

(2) *Library.*—I shall be glad to know the best books to select for a new nurse's library?—*Ignoramus.*

(3) *Lecturing.*—Information about qualifications for C.C. lectures wanted.—*Sister*

(4) *Disinfection.*—What kind of carboiic is used for disinfecting?—*Inquirer.*

(5) *Training.*—Kindly tell me to what Liverpool hospitals training schools are attached?—*Thyra.*

### Answers.

(1) *Sanitation* (*Country Bumpkin*).—Write for information about correspondence class to Miss Lamport, 55, Burton Crescent, London.

(2) *Library* (*Ignoramus*).—Write for catalogue to Manager, Scientific Press, 428, Strand, London. If yon make a list from it, and like to submit the seiected names to us, we will advise you with pleasure.

(3) *Lecturing* (*Sister*).—See answer to No. 1.

(4) *Disinfection* (*Inquirer*).—Your question about carbolic and your other queries are exhaustively dealt with in "Sanitation for Nurses," on which a series of articles appeared in The Hospital beginning April 15th, 1893.

(5) *Training* (*Thyra*).—See Burdett's "Hospital Annual," published 428, Strand, London; price 5s.

### The German Hospital, Dalston.

Both trains and tramcars run within a convenient distance of the German Hospital at Dalston, and on leaving either of these conveyances, with their essentially English accessories and occupants, we were hardly prepared to find ourselves within a few minutes actually in the midst of foreign life.

Iron gates enclosed the grounds, which were large enough to permit plenty of air to circulate round the high deep-red brick building. The porter who emerged from the lodge had a pleasant voice and manner, but the English phrases which he uttered so correctly had a foreign accent about them. He indicated the front door with courteous alacrity, and the Matron soon joined us in the waiting-room. She and the sisters, of whom there was one in each ward, belong to a German order of deaconesses.

There was a quaint old-world atmosphere about the quiet home-like edifice, and the intelligent young conductress who showed us each ward introduced us (speaking German) as "English ladies," which somehow in this prosaic northern suburb suddenly converted us, and not our entertainers, into foreigners. We fancied ourselves in Germany, for there were stoves instead of open fires; and the mottoes and the rules were all in that language. The sisters, in their plain white caps and smoothly-banded hair, had all been there for many years. In the male wards a man works under the sister, and helps her in all ways. These attendants or nurses have been for the most part trained in Germany, and give valuable aid. Contentment, courtesy, and kindness met us on every side, and we shall be glad to hear that commercial depressions will not long be permitted to affect the funds necessary to maintain the hospital, and its neighbouring as well as seaside convalescent Homes.

### Primitive Massage.

A correspondent sends us the following interesting paragraph from "Captain Cook's Third Voyage," dated from Society Islands, September 22nd, 1777:—

"I now returned on board my ship, attended by Otoo's mother, his three sisters, and eight more women. At first I thought this numerous train of females came into my boat with no other intention than to get a passage to Matavia, but when we arrived at the ship they told me that they intended to pass the night on board for the express purpose of undertaking the cure of a disorder I had complained of, which was a pain of the rheumatic kind. I accepted the friendly offer, had a bed spread for them on the cabin floor, and submitted myself to their direction. They began to squeeze me with both hands from head to foot, but particularly on the parts where the pain was lodged, till they made my bones crack, and my flesh became a perfect mummy. In short, after undergoing this discipline for about a quarter of an hour I was glad to get away from them. However, the operation gave me immediate relief, which encouraged me to try another rubbing down before I went to bed; and it was so effectual that I found myself pretty easy all the night after. My female physicians repeated their prescription the next morning before they went on shore, and again in the evening, when they returned on board, after which I found the pains entirely removed, and the cure being perfected, they took their leave of me the following morning. This operation is universally practised amongst these islanders; but sometimes performed by the men, but more generally by the women."

### Novelties for Nurses.

### NATURE-FORM SHOES.

(Messrs. Holden Brothers, 223, Regent Street.)

A considerable tendency on the part of the public has evinced itself for some time past to have none of the fashionable pointed shoe gear, but to adopt a more natural form of covering for the foot. Many a would-be purchaser of shoes formed for feet as nature fashioned them has to discover with regret that custom has established a deformity which no resort to clothing of a natural shape will remedy in later life. It is still possible to provide that the feet of the younger generation shall have a chance of better things. Those hygienically inclined will be glad to learn that Messrs. Holden especially provide for the wants of children, and that foot-gear of the most approved scientific principles is made of excellent quality for them.

☛ For Muses' Looking-Glass, Book Reviews, Everybody's Opinion, and Reading to the Sick, see page clxix. et seq.

### The Muses' Looking Glass.

### THE "OLD MASTERS" AT BURLINGTON HOUSE.

We were particularly struck in this, the twenty-fifth exhibition of pictures by old masters, with the general excellence and beauty comprised in the British section. Here are fonnd several representative works of Gainsborough, Reynolds, and Constable, amongst which we are greeted by many an old familiar face, in the portraits of graceful, beautiful women of the past times—faces which, unhappily, are little met with in the life of to-day; but are rather characteristic of the gentle, more womanly dignity of our grandmother's time. This year the collection of Sir Joshua Reynolds is, perhaps, especially remarkable; amongst which his celebrated "Mrs. Carnac" (No. 131), painted in 1778, well known to us all, and "Mrs. Powys and Daughter," a simple, graceful study of semi-classic draped figures, are especially noticeable. In judging Gainsborough's "Honourable Mrs. Thicknesse," we are induced to criticise the painting more as a personality than as a portrait, so vivid and lifelike is the representation. This picture (No. 101) is given, and with reason, a prominent position in the gallery, Gainsborough has been charged with rather subordinating truthfulness to his subject to a certain poetic mannerism of style; in the portrait of "Mrs. Thicknesse" we certainly do not find it to be the case. There are one or two Romneys scattered on the gallery walls, though, perhaps, with the exception of "Mrs. Van der Gucht" (No. 140), none are quite representative of his work.

Whilst we can all claim a familiarity with the works of our own great painters in this collection, one exception is very evident in the case of Hogarth. Here we see for the first time a picture of his which is new to the public, since it has never been exhibited before; neither in the catalogues of the artist's work is any mention made of its existence. This canvas, 49 by 40 inches, represents "The Wedding of Mr. Beckingham and Miss Corbett"—a quaint and humorous representation it is, too, of a very unpretentious ceremony. Here we see a curious bride and bridegroom and a handful of stiffly-standing guests in an unromantic church, the lines of which edifice are in sadly-distorted perspective. The picture does not show the artist at his best by any means, yet all over it is stamped with a certain quiet humour, a minutiæ of detail characteristic of the Hogarthian brush. The fourth room predominates with early Italian pictures, amongst which are many attractive and beautiful specimens; the fifth gallery is devoted to Pettie, the water-colour room to Stothard, the black and white to Blake. Of special interest always are the pictures of younger members among the "Old Masters." "The End of the Harvest" (No. 3) is the best and strongest specimen of these latter, in landscape anyhow; a general sense of breadth and a poetic interpretation of nature stamps the work of Paul Chalmers, the artist. These qualities we find absent in the work of Fred Walter, another representative of the younger generation. In his picture of "The Plough" (No. 8) and the "Wayfarers" (No. 44) it is especially obvious that the reverse holds good in his case. For instance, in his former work, "The Plough," which catches one's eye directly one enters the gallery, there is a sad want of uniformity; his canvas suggests to one that it was painted at different times under different conditions, and, if one may so express it, as if the artist had two focuses of sight. This gives to the general effect, of an otherwise striking study, a general patchiness which is distracting as well as unpleasant.

### THE EXHIBITION OF EARLY ITALIAN ART.

It must not for a moment be supposed that the collection now on view at the New Gallery is formed exclusively of early Italian pictorial art. The exhibition embraces all the manner of arts which flourished in Italy at perhaps the most interesting period of her history. Therefore, with a background of saints and Madonnas, who gaze out upon us with serene eyes from their golden frames, are cases, the beautifully-arranged contents of which consist of rare specimens of bronzes, personal ornaments, plaquettes, cameos, crystals, carvings, embroideries, laces, armour, musical instruments, medals, majolica ware, and last, but far from least, those glorious illuminated manuscripts and breveries which, monuments of skilful handiwork and patience as they are, alike arouse our wonder and admiration. But, first, if we would view pictures and treasures aright, we should turn our attention to the vision of Madonnas and the Holy Child which form the very atmosphere within the walls of the New Gallery, and shut out the world of omnibuses and the noises and nineteenth-century life of Regent Street, and gradually attune us to all that was worthiest and inspiring in the Italy of the thirteenth to sixteenth centuries. We feel then how Art almost wholly lent herself as the exponent of religion—except when the warm southern blood, breaking from the binding chains of conventional demand, turned to hunting, war, or feasting, with a certain lavishness of detail which showed no distaste on the part of the artist for his subject. Yet the Church was the great patron and controller of art, and so countless Madonnas and saints adorn the walls of the after generations, who stand and gaze and, admitting the inimitable religious sentiment breathing from the canvass of such as Fillipo Lippi, Fra Angelico, and Boticelli, wonder why religion has departed from the realms of pictorial art. It is not always that those accustomed to the more voluptuous treatment of a Doré can see at a first glance the spirit that emanates from what appear at first glance to be almost insipid Madonnas and quaint babies of the early Italian painters. But wait awhile; if you have not become friends with many a mild Madonna and infant Jesus so readily to be seen within our own National Gallery, or in other collections of pictures at home and abroad, lose no time, but hasten before the present exhibition is scattered once again amongst the fortunate possessors, and gaze until the true spirit of the religious in art has found acknowledgment at your hands. No painter, perhaps, excelled Boticelli in power of expressing that holy, meek, and wrapt expression so in accord with our ideal of the Virgin Mother, whilst his serene and thoughtful representations of the Holy Babe appeal warmly to our sympathy. There are in the collection over 30 of Boticelli's pictures, and we are able to study the versatility of this eminent pupil of the great master Fillipo Lippi, and whilst we become well acquainted with his mannerisms, we also learn to admire the fidelity to an ideal of religious feeling which he evidently was always striving to attain in his canvases. We have dwelt long on Boticelli considering the many rivals to our attention which hang around. The collection is admirable and representative of all the best in early Italian art—beautiful specimens from the brush of Cimabue, the patron of the greater Giotto, to the later prince of painters, Raphael (who is largely represented), and yet later masters of painting. In the gallery hang drawings, not less beautiful than the pictures for which they served as studies—drawings on soft brown, blue, and creamy grounds, such as artists in the present day may well look at with envious eyes.

Such are the pictures, dwelt on in the most sketchy and inadequate manner, for we hope the pictures will speak to our readers themselves; if not, no words of ours can convey them. Even as it is we have outrun our space, and the art treasures, other than pictures, must be left also to speak for themselves. Vases of carved rock crystal, beautiful beyond description, and dainty as modern Venetian glass, we cannot pass over. The wondrous iron and bronze work calls forth the feeling that truly the early Italians were a nation of artists, whose workmanship ennobled the labour of their hands.

### The Book World for Women and Nurses.

[We invite Correspondence, Criticism, Enquiries, and Notes on Books likely to interest Women and Nurses. Address, Editor, The Hospital (Nurses' Book World), 428, Strand, W.C.]

Catriona: A Sequel to Kidnapped. By Robert Louis Stevenson. (Cassell and Co., London.)

Sequels are proverbially disappointing, but this is evidently one of those exceptions that goes to prove the rule. There is none of the usual bolstering-up of the former "dead and done for" characters in order to make them seem sufficiently alive to last through a fresh volume. Those who are allowed to reappear have quite sufficient vitality left to carry them successfully to the end of the one volume of which the book consists.

David Balfour continues his autobiography, and the reader is in turn introduced to the household of the Lord Advocate of Edinburgh, with its curious mixture of goodwill and sinister intrigue, and then has to follow the hero into captivity upon the Bass Rock, where he was smuggled and successfully hidden until too late to give his evidence in a political murder case.

The Campbells were at that time all-powerful in Scotland (it was soon after the '45), and an offence to any member of their widespread clan was severely punished. If it were the real offender who got the punishment, so much the better; if not, anyone else to whom the head of the clan had a dislike seems to have answered the purpose equally well—of course, all being done with an imposing show of law and order.

In spite of his detention on the Bass, David manages to put in an appearance at the end of the trial, and does enough to satisfy his conscience; and after a few weeks of Edinburgh society, under the care of the beautiful Miss Grants (whose interest in him extends even to the colour with which he is to tie his hair), he starts for Leyden to study for the Bar.

Alan Breck, one of the hunted Stewarts, and a good friend to David, gives him a lively account of how he has to pass his days hidden inside a haystack to avoid being taken. Fortunately he has humour and imagination enough to keep himself alive through the darkness and loneliness of his prison; and he tells his friend David how he sometimes whiles away the time by composing songs and by inventing 'grand springs" for his pipes, and by sometimes playing knucklebones, though "It's a poor piece of business playing with naebody to admire ye." He gives his friend, who has been priding himself on his superior penetration in having evaded the watchfulness of the Macgregors, who are dogging him to murder him, a quiet and clever set down, by telling him that his elation at his safety is due to ignorance rather than to security. "That is the strange thing about you folk of the college learning; ye're ignorant, and ye cannae see it. Wae's me for my Greek and Hebrew; but, man, I ken that I dinnae ken them; there's the differ of it. Now, here's you. Ye lie on your wame a bittie in the bield of this wood, and ye tell me that ye're cuist off these Frasers and Macgregors. Why! 'Because I couldnae see them,' says you. Ye blockhead, that's their livelihood."

Scotland seems too full of dangers to be a comfortable resting-place for our hero, and we are glad to see him landed safely in Holland; here, with Catriona, who seems formed to be "the ideal woman of a young man's dream," he goes through many more perils and adventures, and for some time has to pass as Catriona's brother. At last matters are arranged; the hero and heroine are duly married, and return to Scotland.

The description of the last days of James More, Catriona's unworthy father, dying an exile in Paris, is very vividly written; he was one of those vain, boastful, shifty men, ready to sell himself or anybody else for money; and his daughter's steadfast, pure character stands out all the more vividly in contrast.

Mr. Stevenson's English has become a proverb, but here we hear little of it, for the characters mostly talk Scotch or else last century English. To the reader with a liking for the study of words, it will be interesting to notice how many of the old Scotch words are but applied French ones; "rivers" for provisions, and "gigot," the usual word for our familiar leg of mutton, being amongst them.

Molly and Her Man of War. By Dr. Arabella Kenealy. (Bentley. 6s.)

"Molly and Her Man of War" has no plot to speak of; it is simply the account, by one of themselves, of the adventures of a pair of globe-trotting young women, on flirtation bent, who in the course of their travels make the acquaintance of a captain in the American Navy. To his attractions the fascinating Molly at length succumbs. Molly herself is a very charming young person, whom it is impossible not to like. She is supposed, by the way, to be an artist, but is certainly not the sort of girl generally associated with artistic achievements, unless it be with those triumphs of millinery and Parisian "creations" which lay claim to the title. There is something rather winsome about her intense impudence, especially when she plays the part of cicerone on the gunboat "Terrapin," a scene which is perhaps the most amusing in the book. The "guide book" portion of the volume is (wisely) not allowed to assume too prominent a position; and Naples, Genoa, Leghorn, and Gibraltar simply serve as backgrounds for the adventures and vagaries of Molly and Nan. The quarantine episode at Gibraltar—which, as well as the subsequent voyage to New York, was an incident of Miss Kenealy's own adventures—is amusing enough to read about but was probably the reverse of amusing to experience. In fact, quarantine regulations in general come in for a good deal of ridicule from Miss Kenealy, and certainly appear, in the South of Europe, at least, to be more productive of plague than profit. Apparently Miss Kenealy made use of her enforced stay in the United States to perfect herself in the peculiarities of the American language; but it would have been kinder to her English readers had she furnished a glossary of some of the American idioms employed. What, for instance, is a "regular bed-rock sort of an affair?" How does one set about "mopping up the sidewalks?" and what is the exact nature of the figurative process of "seeing snakes in your boots?" Further, are there any whales in the Gulf of Lyons? Molly and Nan saw some, or said they did; but as the book is described on the title-page as being "more or less veracious," we must conclude that it is sometimes rather less than more so!

---

The English Illustrated Magazine for January contains a pretty fancy story by Carmen Sylva, Queen of Roumania. The story of the snow maiden and her witch mother is gracefully told, after the fashion of the gifted Queen. Alba, the snow maiden, living isolated and alone with her parent, whose occupation it is to spin the gold which eventually found its way into the veil to grace the heads of Roumanian maidens on their marriage day. Baba Coaja, the witch mother, guarded her daughter carefully from the outside world, but, nevertheless, the inevitable Prince of the fairy tale comes on the scene to spoil all the plans made, and his advent is the beginning of the doom which follows Alba's footsteps, and ends in her downfall. Besides this story there are in this number of the Illustrated several others of a highly imaginative order. "An Important Man," a dreamland story, is most originally illustrated, and the transparent nature of the astral body is better conveyed than we remember to have seen it before. Turning from fiction, we find an interesting article by Sir Edwin Arnold on "Indian Viceroys"; a beautifully-illustrated one on "Many Favourites at the Zoo"; and, lastly, one on "French Musical Act," which easily and pleasantly introduces English readers to a field very little known to them generally, namely, modern French composers. It is so usual to regard French compositions of the light and fantastic order, that many gems, both of instrumental and vocal music, are lost to English lovers of music in consequence. The article we have before us introduces us personally to composers, and we shall be the gainers if we go a step farther and learn something of their works for ourselves.

### Everybody's Opinion.

[Correspondence on all subjects is invited, but we cannot in any way be responsible for the opinions expressed by our correspondents. No communications can be entertained if the name and address of the correspondent is not given, or unless one side of the paper only be written on.]

### A DEMAND FOR FAIR PAY.

"A CONSTANT READER" writes: Could the members of the Paddington Vestry really for one moment seriously believe they would be able to secure the services of a thoroughly competent lady sanitary inspector for £60 per annum? THE HOSPITAL clearly points out the position is not one every woman can fill efficiently. It requires special education, much tact, and intelligence, and unless the vestries are willing to pay fairly for this very important and responsible work they will discover that only incompetent women without any definite idea of the duties required at their hands become applicants for the appointments. How can a woman devote her whole time to the work of sanitation and live on £60 per annum? Rather over 4s. daily (six days to the week) is the value thus put upon an educated woman's time; yet a vestry is supposed to be composed of intelligent business men who discourage "sweating" in any form. Recently daily papers have informed the public 5d. an hour was being paid to "the unemployed" by the London vestries for clearing the snow from the streets of the City. Doubtless the work was laborious, but it required no especial skill. An eight-hour day meant 3s. 4d. earned by the roughest of work. Thus to offer to an educated, specially qualified woman only 8d. per day more than is given to a labourer seems proof that the work required from a sanitary inspector has not yet received the consideration to which it is entitled. The vestry should consider the question from a woman's point of view. Their inspector must reside in a respectable locality, and in London the rent of one room only, is a heavy item; coals, gas, food, laundress. clothing, and perhaps it would not always be possible to walk daily to work. After these imperative expenses are deducted how much will remain to the poor inspector? At what rate will she be remunerated for work that demands knowledge, tact, and more than average intelligence if the scheme is to be carried to a successful issue. People are urged, and rightly, to make provision against "the rainy day," but while appointments open to women are so badly paid, all they are able to do is to make both ends meet, and trust the future will be more propitious than the present. The vexed question of the future, when working is no longer possible, mars many a woman's happiness, if not her usefulness. Philanthropic people generously found homes for "decayed gentlewomen," elderly governesses, &c., and they have proved inestimable boons to many, but women of to-day want to be justly paid for their services. If they do work equal in quality and quantity to a man, why should they be only paid half the amount claimed by men as their lawful due? Is it too much to ask that work acknowledged to be valuable should be compensated at such a rate that a woman may live comfortably (not luxuriously) and be able to make provision for the day when she is no longer able to carry on her daily work, instead of having to deny herself every ordinary comfort, even whilst fearing that the workhouse may be her only refuge in her latter days?

### NURSING SMALL-POX.

"A NURSE" writes: So many people shrink away from the mere thought of nursing small-pox that it should be reassuring to the nursing world to read the encouraging report of the Medical Superintendent of the small-pox hospital ships. He considers that when properly protected by vaccination the attendants run but little risk. Out of 1,201 persons employed during the last eight years only six contracted the disease, and all recovered.

### For Reading to the Sick.

"GOD is the best bed-maker; He promiseth to make all a man's bed in his sickness. Herein His art is excellent, not fitting the bed to the person, but the person to the bed."

—"QUAINT COUNSELS" OF THOMAS FULLER.

"O Lord, my God, do Thou Thy holy will—
I will lie still;
I will not stir lest I forsake Thine arm,
And break the charm
Which lulls me clinging to my Father's breast,
In perfect rest.

"They say, who know the life divine,
And upward gaze with eagle eyne,
That by each golden crown on high,
Rich with celestial jewelry;
Which for our Lord's redeemed is set,
There hangs a radiant coronet,
Prepared for virgin souls, and them
Who seek the martyr's diadem.
Nor deem, who to that bliss aspire,
Must win their way through blood and fire;
The writhings of a wounded heart
Are fiercer than a foeman's dart.
Oft in Life's stillest shade reclining,
In desolation unrepining,
Without a hope on earth to find
A mirror in an answering mind.
Meek souls there are, who little dream
Their daily strife an angel's theme,
Or that the rod they take so calm
Shall prove in Heaven a martyr's palm.

"'O, Father, not My will be done,'
So spake the Son.
Be this our charm, mellowing earthly ruder noise
Of griefs and joys:
That we may cling forever to Thy breast
In perfect rest!"

—KEBLE'S "CHRISTIAN YEAR."

Suffering, allied as it is in some mysterious way to sin, is also closely connected with the sanctification of the faithful. Christ, the true Vine, says of each living branch, "He purgeth it, that it may bring forth more fruit." Nor is it difficult to see how this may be, for the afflicted servant of Christ, drawn apart from the turmoil of the world, ever looking upward to his dear Lord's cross, watching His face of sorrow, "marred more than any man's," and bearing on His aching brow the Crown of Thorns—such an one, while sitting at Jesus' feet, realises more and more His love in suffering, and discovers thus the secret of bearing patiently his own little cross.

And this close commuuion with Christ in suffering is doing for him what nothing else could have done. Out of that softened heart will spring love, sympathy, patience, hope—yea, all the blessed "fruits of the Spirit," until he in his little measure is like the "Captain of our salvation," being made "perfect through sufferings."

Passing by the more ordinary forms of sickness, let us dwell on those life-long sufferers who have perhaps from their earliest years been chained to some couch of pain, one year following another with little variation. They know it may be that no earthly remedy can bring them deliverance, that none can heal their sore disease. Suffering behind, suffering ever before them, until they shall lay aside the burthen of the flesh, and reach the haven where they would be.

Among such as these God is working manifestly. In loneliness, in silence, during wakeful nights and wearisome days, God is teaching them. They have many a conflict with self, many a fear, but onward they go, winning continually, as God enables them, little victories over self and sin; while learning of Him who was meek and lowly of heart, the impatient spirit gains patience, the self-willed, rebellious temper is gradually overcome, until at length the blessed lesson of self-renunciation—of lying still in God's hands, is learnt almost perfectly, even here.—M. B. B.—FROM "VOICES OF COMFORT."

